# A false positive hair ethylglucuronide dosage in an alcohol abstinent patient after liver transplantation: a case report

**DOI:** 10.3389/fmed.2024.1363012

**Published:** 2024-04-03

**Authors:** Thierry Favrod-Coune, Déborah Lidsky, Julien Vionnet, Giulia Magini, Julien Déglon, Barbara Broers

**Affiliations:** ^1^Hôpitaux Universitaires de Genève (HUG), Genève, Switzerland; ^2^Centre Hospitalier Universitaire Vaudois (CHUV), Lausanne, Switzerland

**Keywords:** alcohol, liver, transplantation, test, ethylglucuronide (ETG)

## Abstract

The use of direct alcohol biomarkers (ethylglucuronide and phosphatidylethanol) has recently been implemented in a clinical setting. Due to their low alcohol detection threshold, high sensitivity, and specificity, these tools are very useful in the pre- and post-liver transplantation setting, where the history and physical signs are not always reliable. However, the interpretation of the results can sometimes be misleading and must be integrated into a global clinical evaluation and, more importantly, in the clinical context of each patient. We present here a case report illustrating a false-positive hair ethylglucuronide caused by the application of a capillary gel in an abstinent patient after liver transplantation. This reminds us that even the most accurate laboratory tests must be interpreted with caution.

## 1 Introduction

The most known and used biomarkers to detect alcohol use are indirect measures, such as transaminases, gamma-glutamyl transferase (GGT), or carbohydrate-deficient transferrin (CDT). They suffer from different methodological limitations: low sensitivity, low specificity, and a limited detection period (1). They can remain in the normal range even in the presence of significant alcohol consumption, or be influenced by other factors, for example, other conditions (cirrhosis), medication, or genetic diversity.

Ethylglucuronide (ETG) and phosphatidylethanol (PEth) are direct alcohol biomarkers. ETG is a minor ethanol metabolite generated in the liver. It can be measured in urine or hair. In urine, the threshold of positivation for ETG is approximately a half-drink per day (5 g of alcohol) and is detectable for 3 to 5 days. A study among liver transplantation (LT) candidates found 89% sensitivity and 99% specificity using a cutoff of 0.5 mg per liter (2). In hair (best obtained from the occipital zone), ETG can be detected for a period of 1 month per 1 cm of hair. Studies have been realized with up to 6 cm of hair, and the accepted thresholds of ETG are less than 7 pg/mg for nondrinkers, 7 to 30 pg/mg for low to excessive alcohol use (10 to 60 g of ethanol per day), and more than 30 pg/mg for heavy drinking (more than 60 g per day) (3). Its sensitivity has been evaluated at approximately 80% because a small alcohol amount can remain undetected, but its specificity is between 94 and 100% (4).

PEth is a phospholipid group built through the action of phospholipase D only in the presence of ethanol. Considering that no contamination happened during sampling, its blood concentration therefore reflects the amount of alcohol consumed, or its absence indicates abstinence. The cutoff of 40 micrograms per liter has recently been accepted. Taken as a dry blood spot on a capillary collection, it is an easy way to obtain a direct and reliable marker for alcohol intake. No enzymatic polymorphism, comorbid illness, or medication has been found to change its blood level. The most often used isoform is the 16:0–18:1 form, which increases approximately 6 h after alcohol ingestion and has a half-life close to 4 days, with detection possible during 2–3 weeks. Its sensitivity and specificity are 86 and 100%, respectively, meaning that a small amount of alcohol (e.g., a few drinks in the whole detection period) can remain undetected (5). Its specificity is close to 100%, which means that false positives are essentially related to the contamination of the samples or a manipulation error in the laboratory.

These tests, formerly used almost exclusively in a legal medicine setting, are now used in the clinical setting, especially in the evaluation of patients suffering from alcohol-related cirrhosis and being evaluated for organ transplantation. This is useful in this setting because alcohol abstinence is warranted and history is not always reliable; one patient out of two has been found to have a positive ETG even when declaring abstinence (6). The proper selection of the patient that will benefit from the graft is essential because the number of people needing it exceeds the availability of organs. Still, for a given patient, the impact of a false-positive result of the ETG or PEth test can be life-threatening since the transplantation can be refused. Furthermore, if during follow-up after transplantation the patient presents a false-positive test, the therapeutic relationship with the caregiver will be negatively impacted, with a potential break in mutual trust.

We hereby present a case report of a patient who presented an elevated ETG sample after a combined kidney and LT, while the patient and his family confirmed total abstinence, consistent with the overall clinical picture.

## 2 Case report

A 48-year-old Caucasian man was referred for alcohol evaluation by a specialist in addiction medicine because of a positive hair ETG sample (more than 300 pg/mg), 1 month after a combined liver and kidney transplantation. This test was performed on a 3.5-cm-long hair sample because of an unexplained GGT elevation that was already present for 6 months before the operation. His medical history included liver cirrhosis due to metabolic syndrome and alcohol toxicity (100 g ethanol per day), end-stage kidney disease under hemodialysis that originated from hepatorenal syndrome, and immunoglobulin A (IgA) nephropathy. The patient was also treated for peritoneal and disseminated tuberculosis with ethambutol and moxifloxacine for 9 months before and after the double transplantation.

Before the LT, he underwent regular pre-LT evaluation, including a full substance use and psychiatric assessment. He met the criteria of alcohol abstinence for more than 6 months, having no unstable psychiatric disease, and having a supportive social environment. In the past, he had been an excessive drinker without meeting the criteria for alcohol dependence. He had been able to stop his alcohol consumption within a few weeks without help, medication, or withdrawal symptoms and remained abstinent from alcohol for 3 years. He did not present with any other substance use disorder.

The transplantation team became preoccupied because of the GGT elevation and an ETG result of more than 600 pg/mg. They suspected an alcohol use relapse, challenging the declarations of the patient and his wife.

The clinical evaluation showed no signs or symptoms of alcohol use (facial erythema, hypertension, and breath analysis), and the patient confirmed he was doing well, abstinent from alcohol, and happy with the recent birth of a grandchild, corroborated by his wife. A precise dietary history allowed for an important daily ingestion of white wine vinegar with raw vegetables. The amount was estimated at approximately 1 tablespoon per day (14.8 mL or 0.5 lf oz).

The other laboratory analyses included liver tests showing elevation of GGT (60 UI/L, 9 months before transplantation, to 174 UI/L just before). The other liver tests (transaminases, alkaline phosphatase, and total bilirubin) remain mostly in the normal range (Table 1). The GGT levels then increased to 331 UI/L and the alkaline phosphatase to 222 UI/L 3 months after the transplantation. Those tests were then normalized 1 year later. Concerning the renal function, the creatinine went from approximately 300 to 400 μmol/L pre-transplantation (on hemodialysis) to 130 to 150 μmol/L 6 months after transplantation.

**Table 1:** Paraclinical tests.

Test		Normal Range	9 months before LT	Pre-LT	Just after LT	3 months after LT	6 months after LT	1 year after LT
Urea	mmol/l	3.2–7.5	–^*^	–^*^	7.0	9.5	12.9	8.9
Creatinine	μmol/l	62–106	–^*^	–^*^	287	177	204	165
Estimated Glomerular Filtration Rate (CKD-EPI)	ml/min/1.73 m^2^	> 60	–^*^	–^*^	21	38	32	41
ASAT	U/l	14–50	37	26	895	36	20	9
ALAT	U/l	12–50	21	18	541	56	18	7
Alcaline phosphatase	U/l	25–102	108	79	33	222	67	83
Gamma-glutamyl transpeptidase (GGT)	U/l	9–40	60	174	42	331	82	48
Ethylglucuronide (ETG)	pg/mg	< 7–30	–	–	> 600	517	< 7	-
Phosphatidylethanol (PEth)	Microg/L	< 20–40	–	–	–	–	< 40	-

^*^Irrelevant because on hemodialysis.

The second hair ETG level 3 months after transplantation was at 517 pg/mg, and the third 6 months after was negative. At that time, a PEth test (not available in our hospital before) was also performed and was negative.

The global clinical evolution 3 years after transplantation was favorable, and the patient remained abstinent from alcohol.

To assess if the wine vinegar could induce a false-positive result for ETG and Peth, a healthy female volunteer (59 years old) took the same amount of white vinegar for 3 months while being abstinent from alcohol. The blood PEth and hair ETG dosages remained negative during the follow-up (data not shown).

## 3 Discussion

In this clinical case, the false-positive ETG result had an important negative impact on the patient, his family, and his relationship with the transplantation team. The addiction specialist concluded that the patient was abstinent from alcohol, having no clinical argument to think otherwise, and that the ETG dosage was an exceptional false positive. Still, the patient and the specialist were constantly challenged by the transplantation team. In such situations where a discrepancy exists between the history and a diagnostic test, we emphasize the importance of going back to the patient and his relatives, taking a more detailed history, and considering a broader differential diagnosis. This is particularly relevant in substance use situations, as patients suffering from dependence might be considered in the first place as unreliable. In this situation, the vinegar intake was not the source of ETG; only homemade vinegar would be susceptible to containing ethanol. The source of ETG turned out to be hair gel.

An ETG result of 300 pg/mg suggests an intake of at least 200 g of alcohol per day, which corresponds to almost 3 bottles of wine daily. This was not compatible with the patient and relatives’ histories, nor with the normal clinical examination. Such a high alcohol intake would have been noticed by the relatives, who had no reason to dissimulate a possible intake since the patient already received the LT. It could also have provoked typical clinical signs, such as hypertension, facial erythema and tumefaction, and inflammation of the liver with an increase in liver enzymes.

Hair ETG has been found in the literature to accumulate in cases of advanced renal insufficiency (7). As a source of ethanol should exist to create ETG, suspicion was raised about a dialysis chemical containing some ethanol. This hypothesis had been invalidated by the nephrology team, but as mentioned before, the patient’s diet was found to contain an uncommon vinegar, which we hypothesize has created the ETG (8). Our simple “control” study with a healthy volunteer taking a similar amount of wine vinegar could not confirm the hypothesis.

The elevation of cholestatic liver enzymes (especially GGT), which originally motivated the ETG dosage, was then attributed to the tuberculosis treatment, which was administered 9 months before and as long after the graft and contained antibiotics known to have hepatic toxicity.

The favorable evolution and the discussion between the transplantation team and the addiction team reassured the former about the patient’s abstinence. This was very important since the patient felt unfairly accused and stigmatized. We could not exclude that the caregivers may have presented judgmental speech or behavior (9). We consider that one of the pivotal roles of the addiction specialist is to serve as an intermediary between the patient and the caregivers in general hospitals, first by facilitating communication and second by educating the teams about the nature of dependence and the prejudices often present in such situations.

## 4 Conclusion

In clinical practice, especially in complex situations such as before transplantation involving substance use, no laboratory finding or toxicologic analysis—even the most reliable ones—should replace the patient history and the clinical examination. Each patient must be considered without judgment and with the basic assumption that their history is reliable. In these situations, as with other health-impacting behaviors, the history of the patient’s relatives is an important resource. In cases of contradiction between the clinical evaluation and laboratory or toxicologic findings, our recommendation is to first repeat the test in order to exclude possible confounders and, secondly, change the type of test (e.g., PEth). A false-positive result should always be considered, even in tests with high specificity. This is of outstanding importance in alcohol consumption evaluation before LT, as a wrong conclusion could lead to the death of the patient.

## Data availability statement

The original contributions presented in the study are included in the article/supplementary material, further inquiries can be directed to the corresponding author.

## Ethics statement

Ethical approval was not required for the study involving humans in accordance with the local legislation and institutional requirements. Written informed consent to participate in this study was not required from the participants or the participants' legal guardians/next of kin in accordance with the national legislation and the institutional requirements. Written informed consent was obtained from the individual(s) for the publication of any potentially identifiable images or data included in this article.

## Author contributions

TF-C: Writing – review & editing, Writing – original draft. DL: Writing – review & editing. JV: Writing – review & editing. GM: Writing – review & editing. JD: Writing – review & editing. BB: Writing – review & editing.
